# Editorial: Regional and molecular fingerprint of adipogenesis in aging and disease

**DOI:** 10.3389/fcell.2022.1095235

**Published:** 2023-01-05

**Authors:** Drenka Trivanović, Rossella Labella, Josefine Tratwal, Diana Bugarski

**Affiliations:** ^1^ Group for Hematology and Stem Cells, Institute for Medical Research, National Institute of Republic of Serbia, University of Belgrade, Belgrade, Serbia; ^2^ Department of Physiology and Cellular Biophysics, Columbia University, New York, NY, United States; ^3^ Laboratory of Regenerative Hematopoiesis, Institute of Bioengineering, Ecole Polytechnique Fédérale de Lausanne (EPFL) and Department of Biomedical Sciences, University of Lausanne (UNIL), Lausanne, Switzerland

**Keywords:** adipose tissue, stem cells, metabolism, inflammation, aging, obesity, cancer, mitochondria

During aging, adipose tissue (AT) undergoes dramatic changes, leading to white AT (WAT) and brown AT (BAT) dysfunction and redistribution, changes in the number and differentiation of adipocyte progenitors and accumulation of inflammatory and senescent cells. Since AT plays an integral role in healthspan, understanding of molecular changes in the AT microenvironment during aging and disease would contribute to fundamental and translational research of AT. This Research Topic has collected five original research and 3 review papers on the AT in aging and pathologies.

Population diversity of AT cells has been recently determined at single cell level (Emont et al., 2022). The pool of AT progenitor/stromal cells (ASCs) within the stromal vascular fraction are source of adipocytes and are crucial for the maintenance of AT different location (subcutaneous, visceral, bone marrow) with distinct functions (WAT and BAT). Profiles of AT progenitors vary according to anatomical and functional apportionment and arrange region-specific AT microenvironments. Therefore, diverse ASCs are included in maintenance of subcutaneous, visceral AT or bone marrow adipose tissue (BMAT), and their cellular and molecular dissection is of paramount interests for understanding of aging-related AT alteration.

Aging is gradual process of changes and age-related AT alternations include reduction in the ASC number and their adipogenic ability. Da Nadyellem Silva and Amato review the origin, the features, and the age-related changes of thermogenic AT. Thermogenic BAT declines with aging due to reduced ASC number, differentiation, mitochondrial function, and altered paracrine and endocrine signals. Although promoting the browning of WAT can be a promising anti-obesity and anti-cancer strategy, molecular targets still need to be identified (Cheng et al., 2019; Wolfrum and Gerhart-Hines, 2022). Aging is not the only process that contributes to the changes in AT. Thus, hypoxia-inducible factor (Hif)-3α expression and methylation are AT depot-specific and related to AT dysfunction in states of inflammation and obesity (Pfeiffer et al., 2016). Moreover, Cuomo et al. proved that Hif3α regulates metabolic reprogramming and BAT induction since Hif3α silencing promotes “browning” of white precursor cells. These findings suggest that modification at transcriptional level can bring certain beneficial effects in preserving BAT functions.

In the bone marrow microenvironment, aging is followed by the expansion of BMAT and often associated with bone diseases, such as osteoporosis. The origin of BMAT is not fully elucidated but it is possible that expansion of BMAT occurs due to uncontrolled differentiation of mesenchymal stromal cells (MSCs). The study by Zhang et al. shows that SUMO specific peptidase (SENP)3 knockdown recovers osteogenic differentiation and impairs undesirable adipogenesis in MSCs of glucocorticoid-induced osteoporosis (GIOP) mice. Thus, SENP3 might be realistic MSC target in GIOP disease and might help to combat BMAT expansion and aged osteoporotic profile prevention. Precise elimination of senescent AT cells by using senolytics, senomorphics or exercise can overcome AT aging, improving insulin sensitivity and extending lifespan. Therefore, we can conclude that there exists certain overlap in the action of anti-aging and the anti-adiposopathy agents.

AT dysfunction (adiposopathy) and obesity are main risk factors for age-related states including diabetes, cancer, and cardiovascular diseases. Together with aging, AT inflammatory microenvironment (metaflammation) is consider as a major denominator of AT-related pathologies. Thus, it is of fundamental importance to elucidate whether inflammaging and metaflammation share common inflammatory pathways. Inflammation in obese AT is marked by multiple inflammatory cytokine production, as well as infiltration of AT monocytes, macrophages (ATMs) Choi et al. and leukocytes, leading to chronic inflammation, dysregulated metabolism and AT remodelling (Kahn et al., 2019). Specific deletion of Connexin 43 (Cx43) in ATMs indicates a protection from diet-induced inflammation Choi et al. Thus, it is speculated that this can be used as therapeutic approach to reduce obesity-associated inflammation. In addition to ATMs, presence of regulatory T cells (Tregs) is implicated in age-related insulin resistance (Bapat et al., 2015). However, inflammation governs adipogenesis. As reported by Cuomo et al., inflammation leads to Hif3α upregulation and finally, dysregulated adipogenesis program. On the other hand, expression of inflammatory markers, such as a major innate immune protein lysozyme (Lyz2) gene sustains adipogenesis Lluch et al. as shown in cell line culture.

As expected, due to specific functions and metabolism, different AT depots respond in distinct ways to inflammatory stimuli. Visceral adipocytes have a lower gene expression of adipogenic markers and a higher expression of immunogenic markers in comparison with subcutaneous adipocytes, where ASCs from both depots of obese women retained their region-specific adipogenic capacity during obesity Mathur et al. Moreover, accumulation of visceral and loosing of subcutaneous AT is a hallmark of aging in humans, where central obesity and increased VAT accumulation are recognized as valuable anatomic diagnostic criterion for metabolic diseases (Bays et al., 2014). Interestingly, aging-associated loss of subcutaneous AT can be regulated by population of aging regulatory cells (ARCs) that inhibit proliferation and differentiation of ASCs (Nguyen et al., 2021) and it will be important to reveal their role in different AT depots. Homeotic genes (Hox) are evolutionary conserved regulators that serve as cellular positional identity markers and are involved in regional distribution and specification of AT. However, the significance of Hox genes in aged and diseased AT properties is yet to investigate. Despite the cell-centric concept of region-specific AT differences, contemporary approaches favour global and systemic assessment of AT functions particularly in pathological states (Bays et al., 2014). Thus, combined and integrated single-cell and multi-regional investigations of AT are required.

Therefore, regulation of ATM, Treg, ARC populations, and inflammatory cues can contribute to conceiving of new treatments for AT disorders, where both global and region-specific AT changes have to be considered.
